# Thin‐layer drying behavior of West Indian lemongrass (*Cymbopogan citratus*) leaves

**DOI:** 10.1002/fsn3.642

**Published:** 2018-04-17

**Authors:** Saheeda Mujaffar, Sherida John

**Affiliations:** ^1^ Food Science and Technology Unit Department of Chemical Engineering The University of the West Indies St. Augustine Trinidad and Tobago

**Keywords:** activation energy, diffusion coefficients, Fick's Law, lemongrass, oven drying, thin‐layer modeling

## Abstract

The objectives of this study were to investigate the effect of temperature (40, 50, 60, and 70°C), and air velocity (0.5, 1, and 2 m/s) on the drying behavior of West Indian lemongrass (*Cymbopogan citratus*) leaves. Drying was carried out in a computer‐controlled tray dryer. Overall, the effect of temperature was seen to be more important than that of air velocity, but the air velocity did have an effect on drying rates at the start of the drying process at 50–70°C. Drying rate constants, diffusivity values, and activation energy were determined. Twenty‐two empirical and semiempirical thin‐layer models were tested, and although model fit varied, the Midilli model could be applied to all data with reasonable prediction of *MR* values.

## 1 INTRODUCTION

Lemongrass is an aromatic grass well known for its medicinal, therapeutic, and flavoring/culinary uses. As the name suggests, the plant is identified by its characteristic light lemon scent due to the volatile oils present in the plant. The herb is very popular in Asian, Thai, and Vietnamese cooking and can be used in the fresh, dried, or powdered forms as seasonings and teas. In the Caribbean, the West Indian lemongrass (*Cymbopogan citratus*) grows easily and the fresh leaves used as a tea to treat fevers, hence, the common name, “fevergrass”. Lemongrass leaves are rich in the aldehyde citral, containing between 78 and 82% (Daniel, 2006). This essential oil has many cosmetic applications and can also be used as an insect repellent.

Drying is a key step in the preservation of lemongrass leaves and in the further processing of the leaves into value‐added products, as well as oil extraction. Oven (cabinet) drying still remains an attractive option for bulk drying of fragrant leaves such as coriander, olive mint, basil, thyme, bay leaves, and olive leaves (Ahmed, Shivhare, & Singh, 2001; Erbay & Icier, 2010; Cakmak, Kumcuoglu, & Tavman, 2013; Rodríguez, Clemente, Sanjuán, & Bon, 2014; Doymaz, 2014). A review of the work performed on lemongrass reveals studies which focus on different aspects of the drying process, including quality and essential oil content (Lonkar, Chavan, Pawar, Bansode, & Amarowicz, 2013; Martinazzo et al., 2009), drying efficiency (Kassem, El‐Batawi, & Sidky, 2006), effect of pretreatments (Lonkar et al., 2013), drying curves (Coradi, Melo, & Rocha, 2014; Fudholi et al., 2012; Ibrahim, Sopian, & Daud, 2009; Rahman, Tasirin, Razak, Mokhtar, & Muslim, 2013), and mathematical modeling of drying data (Coradi et al., 2014; Ibrahim et al., 2009; Kemat, Rahman, & Wahit, 2008; Waewsak, Chindaruksa, & Punlek, 2006). However, with respect to the drying method employed, there are limited studies on lemongrass which include conventional cabinet drying (Kassem et al., 2006; Lonkar et al., 2013). Previous works have instead focused on the drying of leaves in a solar dryer well as other dryers such as fluidized bed dryer, heat pump dryer, constant temperature and humidity chamber, biomass dryer, and a fixed bed dryer (Kassem et al., 2006; Waewsak et al., 2006; Kemat et al., 2008; Ibrahim et al., 2009; Fudholi et al., 2012; Sanmeema, Poomsa‐ad, & Wiseet, 2012; Rahman et al., 2013; Coradi et al., 2014). The effect of drying parameters (such as temperature and velocity) on the behavior of leaves has not been reported for leaves dried in conventional ovens. Work has been reported for leaves dried in a fluidized bed dryer at a range of temperatures but at fixed or small range in air velocity (Kemat et al., 2008; Rahman et al., 2013) or at a range of relative humidity values (Ibrahim et al., 2009). With respect to drying rates, no information has been presented on the effect of drying parameters on moisture curves and drying rate curves for leaves dried in conventional ovens. Drying curves were presented by some authors (Ibrahim et al., 2009; Fudholi et al., 2012; Coradi et al., 2014). Rahman et al. (2013) calculated drying rate of leaves dried in a fluidized bed dryer in units of g/g min. Lastly, with respect to mathematical modeling of drying data, modeling has been performed for methods other than conventional drying, with limited drying variables and using selected thin‐layer models, which vary in their method of model assessment (Coradi et al., 2014; Ibrahim et al., 2009; Kemat et al., 2008; Waewsak et al., 2006).

Due to the gaps in the information available, this work was therefore undertaken to systematically describe the effect of temperature and air velocity on the drying behavior of leaves dried in a conventional, drying oven. This study was conducted to collect baseline drying data for West Indian lemongrass (*Cymbopogan citratus*) leaves dried at three (3) drying air temperatures and velocities, and using two pretreatments. The specific aims were to develop drying curves, determine drying rate constants and diffusion coefficients, and model the drying data using twenty‐two thin‐layer models. Due to experimental limitations in a typical cabinet oven, the relative humidity (*rh*) of the drying air was not controlled, noting as well that humidity is not usually manipulated during a typical drying process.

## 2 MATERIALS AND METHODS

### 2.1 Raw material

Mature West Indian lemongrass (*Cymbopogan citratus*) leaves were sanitized using a 1% bleach (sodium hypochlorite) solution, pat with paper towels to remove excess water (Kadam, Goyal, Singh, & Gupta, 2011), then allowed to air‐dry for approximately 30 min. To fit on the drying trays, the leaves were then cut into pieces 20 cm in length. A total of 4.2 kg of lemongrass was used in the drying experiments.

### 2.2 Drying procedure and treatments

**Figure 1:**
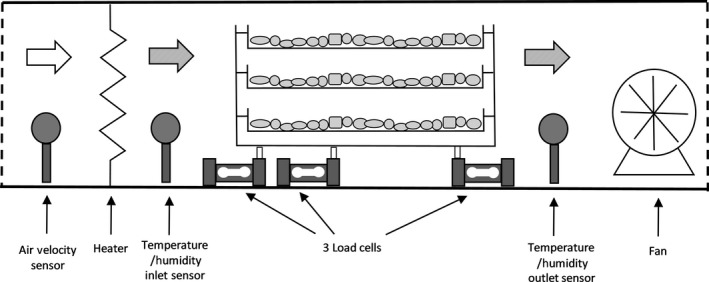
Schematic of Armfield UOP8‐KK11 tray drier

To evaluate the effect of drying air temperature and air velocity, drying was carried out in an Armfield UOP8MK‐II tray dryer (Armfield, Hampshire, England). The dryer temperature (up to 75°C at 0.45 m/s) and air flow rate (0.45 to 1.9 m/s) were computer controlled. The dryer consisted of three removable trays (0.30 × 0.25 × 0.01 m), variable speed fan, and metal louvers to manipulate air velocity, and humidity sensors and load cells (Figure 1). Data logging and analysis software included temperature and air velocity control and data recording.

Approximately 150 g of lemongrass leaves was used for each drying run, averaging 50 g per each drying tray. The leaves were spread onto each of the three drying trays in single layers and weight (g) measurements during the drying process was automatically recorded at 20‐min interval, until there were no further changes in weight. Leaves were then removed from the drier, allowed to cool to ambient temperature (30°C) before storing in resealable plastic bags until analysis. Drying was carried out at four (4) temperatures: 40, 50, 60, and 70°C and at three (3) air velocities: 0.5, 1, and 2 m/s. Drying runs at each air temperature and air velocity were completed in duplicate, so a total of 24 runs were carried out. The average air temperature, relative humidity, and air velocity values recorded in the dryer are given in Table 1.

**Table 1:** Experimental conditions for the drying of lemongrass leaves

Set temperature (°C)	Set air velocity (m/s)	Actual temperature (°C)	Relative humidity (%)	Tray velocity (m/s)
40	0.5	40.0 ± 0.21	39.0 ± 1.62	0.68 ± 0.04
	1.0	40.1 ± 0.39	34.8 ± 2.34	1.06 ± 0.05
	2.0	40.0 ± 0.20	36.3 ± 2.73	1.96 ± 0.07
50	0.5	50.0 ± 0.25	24.6 ± 1.28	0.67 ± 0.04
	1.0	50.2 ± 1.76	23.8 ± 2.11	1.02 ± 0.08
	2.0	50.0 ± 0.31	21.2 ± 1.48	1.83 ± 0.07
60	0.5	59.9 ± 0.39	19.8 ± 1.72	0.51 ± 0.03
	1.0	60.0 ± 0.51	15.0 ± 2.71	1.24 ± 0.08
	2.0	57.7 ± 0.83	17.4 ± 1.44	1.81 ± 0.05
70	0.5	69.7 ± 0.52	13.2 ± 2.36	0.6 ± 0.04
	1.0	69.9 ± 0.52	12.2 ± 1.70	0.9 ± 0.03
	2.0	69.9 ± 0.62	10.6 ± 0.66	1.78 ± 0.05

Preliminary drying runs were conducted to investigate the effect of drying pretreatments used by Lonkar et al. (2013), namely water and chemical blanching (1% sodium carbonate solution, w/v) followed by drying at 50°C (1 m/s). It was found that these pretreatments did not significantly affect the equilibrium moisture values of dried leaves nor the time taken (min) to achieve equilibrium moisture content when compared with untreated leaves. Leaves subjected to pretreatments prior to drying experienced negative changes in color, turning yellow brown on drying.

### 2.3 Analytical methods

To determine the moisture content (wb) of fresh and dried leaves, samples were dried at 103°C using a Mettler Toledo HB43‐S (Mettler‐Toledo, Columbus, Ohio, USA) moisture analyzer and moisture content expressed on a dry weight basis (g H_2_O/g DM). Water activity of fresh and dried samples was determined using the Aqualab CX‐2 water activity meter (Aqualab, Pullman, Washington, USA). Color was assessed using the Konica Minolta CR‐400 Chroma Meter (Konica Minolta Optics Inc., Osaka, Japan). Color was defined in terms of the CIELAB L*, a*, b* color space (Hunterlab, 2008): L* represents lightness (0‐black to 100‐white), a* represents red (positive value) and green (negative value) while b* represents yellow (positive value) and blue (negative value). *Hue angle* (°), *Chroma,* and Total color difference (*ΔE*) between fresh and dried leaves were calculated as given in Equations (2) through (3) (Konica Minolta Sensing Inc., 2003).

(1) $$\text{Hue}=\text{Arc}\tan\left(\frac{b^{*}}{a^{*}}\right)\left(\text{degrees}\right)$$

(2) $$\text{Chroma}=\sqrt{\left({a^{*}}^{2}+\;{b^{*}}^{2}\right)}$$

(3) $$\Delta E=\;\sqrt{{\left({L^{*}}_{0}-L^{*}\right)}^{2}+{\left({a^{*}}_{0}-a^{*}\right)}^{2}+{\left({b^{*}}_{0}-b^{*}\right)}^{2}}.$$

Based on observations during preliminary drying runs, four‐point rating scales were developed to assess the color, texture, and odor of the leaves before and after drying. Color was assessed as 4) bright green 3) dull green 2) light green 1) yellow/brown. Lemongrass odor was rated as 4) strong 3) moderate 2) slight 1) none. Leaf texture after drying was assessed as 4) pliable 3) slightly pliable 2) slightly brittle 1) brittle/breaks easily.

### 2.4 Data analysis

The sample weight data (g) at the end of the drying process and the moisture content of the final dried sample were used to back‐calculate the moisture content of the respective samples at each point during the drying process (Mujaffar & Sankat, 2005, 2015). The drying rate constant (*k*) was determined from a plot of ln *MR* versus time (*t*) based on Equation (4) and the effective moisture diffusivity (*D*_eff_) values calculated using Equation (5) with the thickness of the leaves (2*L*) being 0.5 cm.

(4) $$\text{MR}=\left(M-M_{e}\right)/\left(M_{o}-M_{e}\right)=Ae^{-kt}$$

(5) $$k=\left(\pi^{2}D_{\mathrm{eff}}\right)/4L^{2}$$

For this study, a total of twenty‐two (22) empirical and semiempirical thin‐layer models (Alibas, 2014; Kucuk, Midilli, Kilic, & Dincer, 2014; Silva, Silva, Gama, & Gomes, 2014) were applied to the *MR* data (Table 2). Some models are derived from the original older models. As is now a common practice in thin‐layer drying studies, the performance (fit) of the models was assessed through the use of the coefficient of determination (*R* ^*2*^), root mean square error (*RMSE*), and the chi‐squared statistic (χ^*2*^). Further regression analysis and ANOVA were carried out using GenStat for Windows Discovery Edition 4 Software (VSN International Ltd., 2014). Model fit was carried out using Curve Expert Professional software, version 2.3.0 (Hyams, 2016).

**Table 2:** Thin‐layer drying models

Model name	Equation
Newton	MR* = *exp *(−kt)*
Page	MR* = *exp*(−kt* ^*n*^ *)*
Modified Page	MR* = *exp*(−kt)* ^*n*^
Henderson and Pabis	MR* = a* exp *(−kt)*
Modified Henderson and Pabis	MR* = a* exp *(−kt)* + *b* exp *(−gt)* + *c* exp *(−ht)*
Logarithmic	MR* = a* exp *(−kt)* + *c*
Two‐term	MR* = a* exp *(−k*_*0*_ *t)* + *b* exp *(−k*_*1*_ *t)*
Two‐term exponential	MR* = a* exp *(−k t)* + *(1−a)* exp *(−k a t)*
Wang & Singh	MR* = 1 + at* + *bt* ^*2*^
Verma	MR* = a* exp*(−kt)* + *(1−a)* exp*(−gt)*
Hii	MR* = a* exp*(−kt* ^*n*^ *)* + *c* exp*(−gt* ^*n*^ *)*
Midilli	MR* = a* exp *(−kt* ^*n*^ *)* + *b t*
Peleg	MR* = 1 – (x/(a* + *bx))*
Weibull distribution	MR* = a – b* exp *(−kt* ^*n*^ *)*
Diffusion approach	MR* = a* exp*(−kt)* + *(1−a)* exp*(−kbt)*
Aghbashlo et al.	MR* = −k*_*1*_ *t / (1 + k*_*2*_ *t)*
Logistic	MR* = a*_*0*_ */ ((1 + a* exp *(kt))*
Jena and Das	MR* = a* exp *(−kt* + *b t* ^*1/2*^ *)* + *c*
Demir et al.	MR* = a* exp *(−kt* ^*n*^ *)* + *c*
Simplified Fick's Diffusion (SFFD) equation	MR* = a* exp *(−c (t/L* ^*2*^ *))*
Modified Page Equation‐II	MR* = *exp *(−k (t/L* ^*2*^ *))* ^*n*^
Alibas	MR* = a* exp *(−kt* ^*n*^ + *b t)* + *g*

## 3 RESULTS AND DISCUSSION

### 3.1 Quality attributes

**Figure 2:**
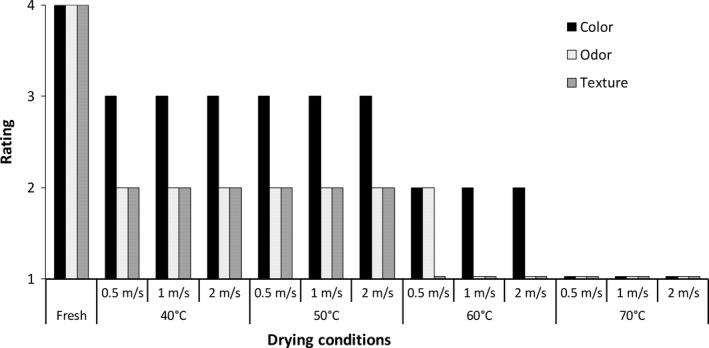
Subjective quality assessment of dried lemongrass leaves. Color 4) bright green 3) dull green 2) light green 1) yellow/brown. Lemongrass odor: 4) strong 3) moderate 2) slight 1) none. Leaf texture: 4) pliable 3) slightly pliable 2) slightly brittle 1) brittle/breaks easily

Fresh leaves were bright green in color (rating of 4) with a strong lemongrass scent (rating of 4) and very pliable in texture (rating of 4). Figure 2 shows that with respect to the subjective ratings for dried leaves, all ratings (color, odor, and texture) were lower than those for the fresh leaves. Leaves dried at 40 and 50°C were similar and showed the least deterioration in quality attributes, while those dried at 70°C showed the greatest deterioration. At the end of drying, leaves dried at 40 and 50°C were dull green in color, while leaves dried at 60°C were lighter. Leaves dried at 70°C appeared yellow/brown. With respect to odor, leaves dried at all temperatures and air velocities displayed a decrease in the characteristic lemon scent of lemongrass having mild to no odor after drying. Leaves dried at lower temperatures retained their aroma better than those dried at 60 and 70°C. Leaves dried at 70°C at all three air velocities had no traces of the lemon scent. Leaves dried at 60 and 70°C leaves were brittle and broke easily. It was also noted that leaves tended to be blown about as drying proceeded at the highest air velocity of 2.0 m/s, as the leaves became less moist and lighter.

The color attributes of fresh and dried leaves are given in Table 3. The L*, *a* ^*^, and *b* ^*^ values of fresh lemongrass leaves averaged 44.23, −10.40, and 12.64, respectively. Compared to the fresh leaves, all attributes were all significantly different in the dried leaves (*p *≤ .05); however, it was difficult to see clear trends with respect to increasing temperature and air velocity. There was an increase in lightness (*L* ^*^ values) when lemongrass leaves were dried. All dried lemongrass samples had negative *a* ^*^ values indicating retention of the green color; however, the values of dried leaves were less than fresh leaves indicating a decrease in “greenness.” There was a small reduction in *b* ^*^ values when leaves were dried. There was a reduction in *Hue (°)* values when leaves were dried, decreasing from −50.47 to an average value of −62.30°, indicating a color shift in the dried leaves. *Chroma* values were decreased from 16.38 in fresh leaves to an average of 12.25 in dried leaves, indicating a decrease in color intensity on drying. Color difference (ΔE) values for dried leaves averaged 6.6, indicating that the difference in color would be perceptible at a glance. This result is interesting, given the large perceptible difference in color as reported in the subjective color ratings for leaves dried at 60 and 70°C. Color difference is not always a suitable criterion by itself and may require further analysis of the individual color attributes (Hunterlab, 2008).

**Table 3:** Color attributes of fresh and dried lemongrass leaves

Air temperature (°C)	Air velocity m/s	Color attribute			Hue (°)	Chroma	ΔE
		*L* ^*^	*a* ^*^	*b* ^*^			
FRESH	NA	44.23 ± 0.55^d^	−10.4 ± 0.24^f^	12.64 ± 0.47^a^	−50.47 ± 0.94^a^	16.38 ± 0.459^a^	REF
40	0.5	50.08 ± 0.98^a^	−4.9 ± 0.3^ab^	9.53 ± 0.06^d^	−62.81 ± 1.28^c^	10.72 ± 0.19^d^	8.63 ± 0.87^a^
	1.0	48.23 ± 2.24^ab^	−5.72 ± 0.42^bcd^	11.26 ± 0.63^bc^	−63 ± 2.97^c^	12.64 ± 0.373^b^	6.57 ± 1.53^bc^
	2.0	48.1 ± 0.485^ab^	−6.95 ± 0.05^e^	11.08 ± 0.56^bd^	−57.88 ± 1.14^b^	13.08 ± 0.50^b^	5.45 ± 0.53^c^
50	0.5	49.32 ± 0.03^ab^	−5.96 ± 0.05^ce^	11.74 ± 0.37^ab^	−63.08 ± 0.90^c^	13.17 ± 0.31^b^	6.83 ± 0.001^ac^
	1.0	48.74 ± 0.44^ab^	−5.78 ± 0.65^bcd^	11.24 ± 0.1^bc^	−62.93 ± 2.81^c^	12.64 ± 0.205^b^	6.68 ± 0.13^ac^
	2.0	49.34 ± 0.64^ab^	−5.78 ± 0.25^bcd^	11.01 ± 0.11^bd^	−62.32 ± 1.24^bc^	12.44 ± 0.02^bc^	7.11 ± 0.33^ac^
60	0.5	45.3 ± 0.39 ^cd^	−5.04 ± 0.03^ac^	9.80 ± 0.515 ^cd^	−62.74 ± 1.34^c^	11.02 ± 0.446 ^cd^	6.20 ± 0.28^bc^
	1.0	46.8 ± 0.68^bc^	−6.05 ± 0.14^de^	10.48 ± 0.32^bd^	−60 ± 0.184^bc^	12.1 ± 0.347^bd^	5.52 ± 0.55^c^
	2.0	48.35 ± 1.04^ab^	−5.73 ± 0.47^bcd^	10.32 ± 0.115^bd^	−61 ± 2.26^bc^	11.82 ± 0.13^bd^	6.68 ± 0.93^ac^
70	0.5	48.77 ± 0.64^ab^	−5.75 ± 0.04^bcd^	10.38 ± 0.035^bd^	−61.03 ± 0.07^bc^	11.86 ± 0.05^bd^	6.90 ± 0.46^ac^
	1.0	47.36 ± 0.38^bc^	−5.93 ± 0.21 ^cd^	11.56 ± 0.09^ab^	−62.86 ± 0.99^c^	12.99 ± 0.01^b^	5.58 ± 0.36^bc^
	2.0	49.12 ± 0.28^ab^	−4.69 ± 0.25^a^	11.67 ± 0.75^ab^	−68.11 ± 0.24^d^	12.58 ± 0.79^bc^	7.62 ± 0.46^ab^

Values are means ± SEM, *n *= 2 per treatment group.

^a‐f^Means in a column without a common superscript letter differ (*p *<* *.05) as analyzed by two‐way ANOVA and the LSD test.

The loss of quality in oven‐dried leaves, especially at higher temperatures, can be attributed to browning reactions at higher temperatures, a decrease in chlorophyll content and essential oils (Coradi et al., 2014; Chen & Patel, 2008; Kassem et al., 2006; Sanmeema et al., 2012). With respect to the effect of air velocity on quality of dried leaves, Coradi et al. (2014) found that increasing the air velocity from 0.8 to 1.3 m/s did not negatively affect the essential oil content of lemongrass leaves when compared with fresh leaves; however, further increasing the velocity to 1.8 m/s resulted in a statistically significant reduction in oil content. They noted the works of Buggle et al. (1999) who dried lemongrass leaves at 30–90°C and found that the highest oil content of 1.43% was found in leaves dried at 50°C. Drying at 30°C favored fungal growth and drying at 70°C resulted in significant reduction in essential oil content to 0.34%. Lightening of leaves after drying has also been reported for coriander and fenugreek leaves (Naidu et al., 2012; Shaw, Meda, Tabil, & Opoku, 2007). High temperature could lead to the replacement of magnesium ions in chlorophyll by hydrogen therefore converting chlorophyll to pheophytins (Naidu et al., 2012). Some studies showed that drying temperature had no significant effect on the color of dried mint and bay leaves (Cakmak et al., 2013; Demir, Gunhan, Yagcioglu, & Degirmencioglu, 2004; Kadam et al., 2011).

### 3.2 Drying curves

**Figure 3:**
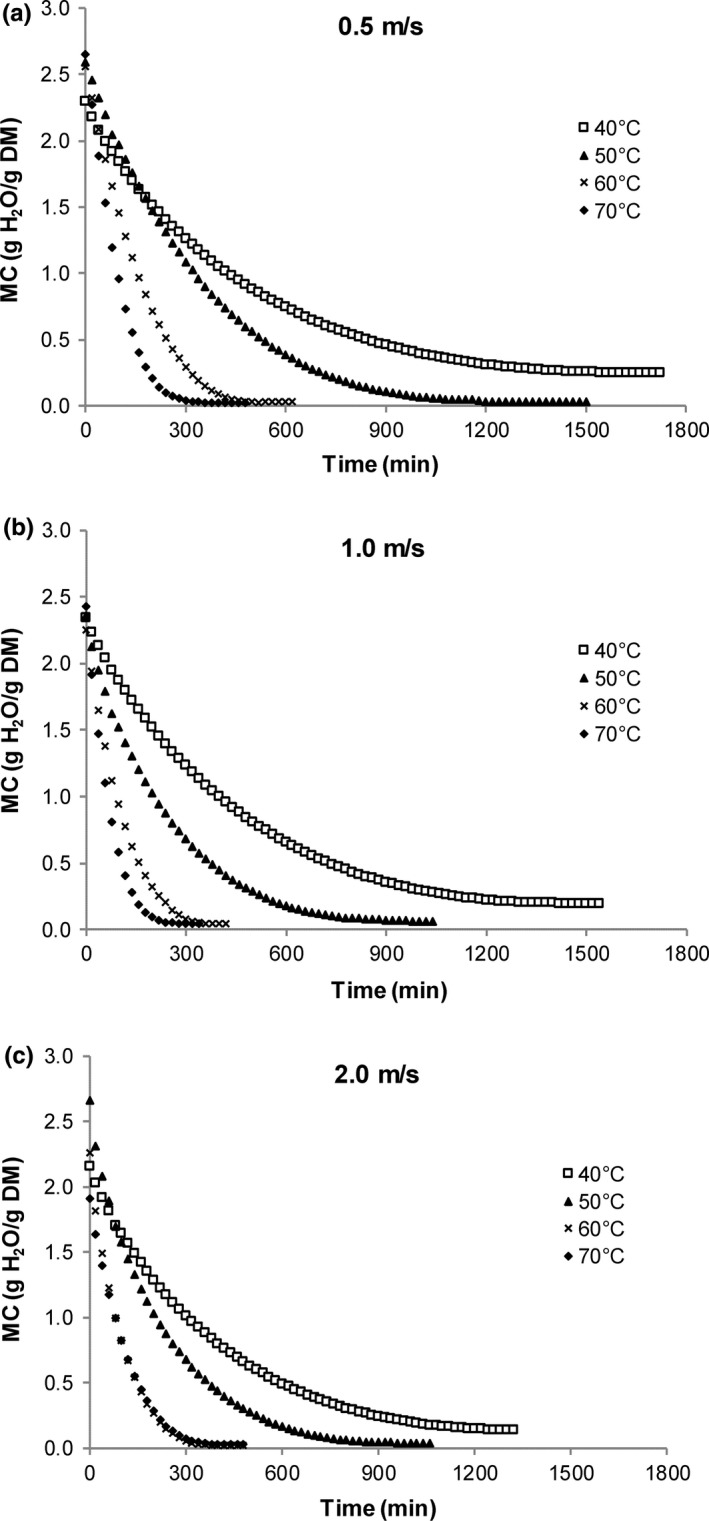
Effect of drying air temperature on moisture content changes in lemongrass leaves dried at different air velocities

**Figure 4:**
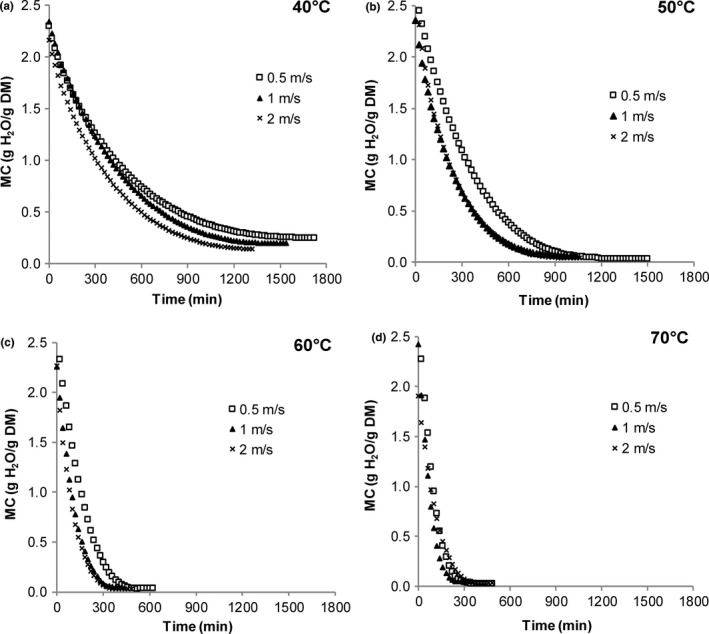
Effect of air velocity on moisture content changes in lemongrass leaves dried at different temperatures

The initial moisture content and water activity values of lemongrass leaves averaged 2.23 ± 0.24 g H_2_O/g DM (69.0% wb) and 0.694 ± 0.009, respectively. Drying curves showing the change in moisture content with time at each air velocity are given in Figure 3a through c. Drying curves showing the change in moisture content with time at each temperature are given in Figure 4a through d. Moisture content during drying was significantly affected by drying time, drying temperature, air velocity, and a time–temperature interaction (*p *≤* *.001). The time–temperature interaction highlights the results that the decline in moisture content will depend on the specific combination of both drying time (min) and air velocity (m/s). During drying, moisture content of leaves was found to decrease in the typical manner, with an initial rapid decline followed by a gradual decrease toward equilibrium.

Generally, for all temperatures and air velocity treatments, the greatest decrease in moisture content occurred during the first 200 min of drying. As temperature was increased from 40 to 70°C at 0.5 and 1.0 m/s, leaves experienced a greater decline in moisture content. This trend was also seen for leaves dried at 2.0 m/s as drying temperature increased from 40 to 60°C, but further increasing the temperature to 70°C did not cause any further change in leaf moisture content (Figure 3c).

As shown in Figure 4a, the effect of increasing air velocity was most apparent at a drying temperature of 40°C, where increasing air velocity resulted in a greater decline in moisture content. At 50 and 60°C (Figure 4b,c), increasing the air velocity from 0.5 to 1.0 resulted in a greater decline in moisture content. However, further increasing to 2 m/s did not result in a further decline in moisture content of the leaves. At the highest temperature of 70°C (Figure 4d), there was a marginal increase in the decline in moisture values of leaves as air velocity increased from 0.5 to 1.0 m/s; however, further increasing the air velocity to 2.0 m/s reversed this trend, with these leaves having similar moisture values to those dried at 0.5 m/s. These results are supported statistically by the temperature interaction effects.

Final equilibrium moisture content and water activity values of dried leaves are given in Table 4. Water activity values for dried lemongrass leaves were in the range 0.566 to 0.571. Overall, the average equilibrium moisture values of leaves dried at 40°C were approximately 80% higher than the average value for leaves dried at higher temperatures. The time taken to reach equilibrium moisture content was significantly (*p *≤ .001) affected by drying air temperature and air velocity, and a temperature‐velocity interaction (*p *≤ .05). Increasing temperature to 50, 60, and 70°C resulted in an average decrease in drying time of 27%, 71%, and 78%, respectively. At fixed air temperature of 40–60°C, increasing air velocity from 0.5 to 1.0 m/s resulted in a significant decrease in drying time, beyond which the effect was not significant. For leaves dried at 70°C, however, the effect of increasing air velocity to 2.0 m/s was reversed, and these leaves took a longer time to dry than leaves dried at 1.0 m/s.

**Table 4:** Moisture, water activity values, and drying times of lemongrass leaves dried under different conditions

Air temperature (°C)	Air velocity (m/s)	Eqm MC (g H_2_O/g DM)	Time to reach eqm (mins)	Final a_w_	Time taken to reach 11% (wb)
40	0.5	0.250 ± 0^a^	1630 ± 190^a^	0.551 ± 0.003^c^	Not achieved
	1.0	0.198 ± 0.022^b^	1390 ± 110^b^	0.569 ± 0.002^b^	Not achieved
	2.0	0.144 ± 0.033^c^	1280 ± 20^b^	0.593 ± 0.002^a^	1260 ± 140^a^
50	0.5	0.039 ± 0.003^d^	1190 ± 10^bc^	0.558 ± 5e‐04^c^	865 ± 35^b^
	1.0	0.044 ± 0.0035^d^	980 ± 100 ^cd^	0.570 ± 0.0015^b^	725 ± 5^bc^
	2.0	0.044 ± 0.004^d^	950 ± 70^d^	0.585 ± 0.0045^a^	665 ± 45^c^
60	0.5	0.032 ± 0.004^d^	530 ± 10^e^	0.556 ± 5e‐04^c^	370 ± 20^d^
	1.0	0.055 ± 0.005^d^	330 ± 10^ef^	0.557 ± 5e‐04^c^	295 ± 5^de^
	2.0	0.025 ± 0.009^d^	390 ± 30^ef^	0.585 ± 0.005^a^	255 ± 25^de^
**70**	0.5	0.030 ± 0.006^d^	330 ± 50^ef^	0.540 ± 0.0015^d^	220 ± 30^e^
	1.0	0.050 ± 0.010^d^	240 ± 40^f^	0.572 ± 0.0035^b^	170 ± 40^e^
	2.0	0.035 ± 0.002^d^	380 ± 60^ef^	0.586 ± 0.004^a^	250 ± 40^de^

Values are means ± SEM, *n *= 2 per treatment group.

^a‐f^Means in a column without a common superscript letter differ (*p *<* *.05) as analyzed by two‐way ANOVA and the LSD test.

Martinazzo et al. (2009) recommended the drying of lemongrass leaves at 50°C to a final moisture content of 11% (wb). As also given in Table 3 for the present study, the time taken for the leaves to achieve an 11% moisture value was significantly affected by temperature and air velocity (*p *≤ .001) and a temperature–velocity interaction (*p *≤ .05). Leaves dried at 40°C at 0.5 and at 1.0 m/s could not be dried to 11% moisture content as equilibrium moisture values averaged 20 and 18% (wb), respectively. Aside from that, the higher the temperature, the shorter the time taken to achieve a 11% moisture value. An increase in air velocity from 0.5 to 2.0 m/s resulted in a shorter drying time at 50 and 60°C. At 70°C, increasing the air velocity from 1.0 to 2.0 m/s did not result in a further decrease in drying time to 11% MC.

Not much has been reported in the research papers on lemongrass on the change in moisture content with drying time, temperature, or air velocity. Fudholi et al. (2012) presented a single drying curve for lemongrass leaves in a solar dryer for a maximum of 6 h. The initial moisture values of leaves were reported to be similar to that in this study, averaging 2.0 g H_2_O/g DM. They reported that lemongrass could be dried from an initial moisture content of 65 to 8% (wb) in 4.5 h. Coradi et al. (2014) presented drying curves at 40–70°C for lemongrass leaves (*Cymbopogan citratus*) dried in a fixed bed dryer with upward air flow, which revealed that increasing air temperature resulted in a greater decline in moisture content beyond 60 min of drying. They reported higher initial moisture content values for fresh leaves (3.12 db %). The time to attain equilibrium at 50°C decreased from 220 min to 190 min as air velocity increased from 0.8 to 1.8 m/s. The shorter drying times compared with this present study could possibly be due to the small sample piece size (2‐cm length) used in that study. Ibrahim et al. (2009) reported that the time required for lemongrass leaves to reach a moisture content of 0.2% dry basis was 550 min at 35°C compared to 200 min at 55°C. Sanmeema et al. (2012) reported that the moisture content of lemongrass leaves (species not given) could be reduced from 180–190% (db) to 10% (db) in a heat pump dryer using hot air at 40–60°C.

The effect of temperature on the moisture content decline of other leafy materials has also been reported for other leafy materials such as coriander, bay leaves, olive, and drumstick (*Moringa oleifera*) leaves (Ahmed et al., 2001; Demir et al., 2004; Doymaz, 2014; Erbay & Icier, 2010; Premi, Sharma, & Upadhyay, 2010). Higher temperatures cause a higher reduction in moisture content as a result of increased heat and mass transfer, which favors evaporation of moisture from the leaves (Aghbashlo, Kianmehr, & Hassan‐Beygi, 2010; Doymaz, 2006). With respect to the effect of air velocity increasing moisture loss up to limiting velocity as found in this study, Tzempelikos, Vouros, Bardakas, Filios, and Margaris (2014) found that increasing air velocity beyond 2 m/s did not effect a further decline in moisture content of quince slices.

### 3.3 Drying rate curves

**Figure 5:**
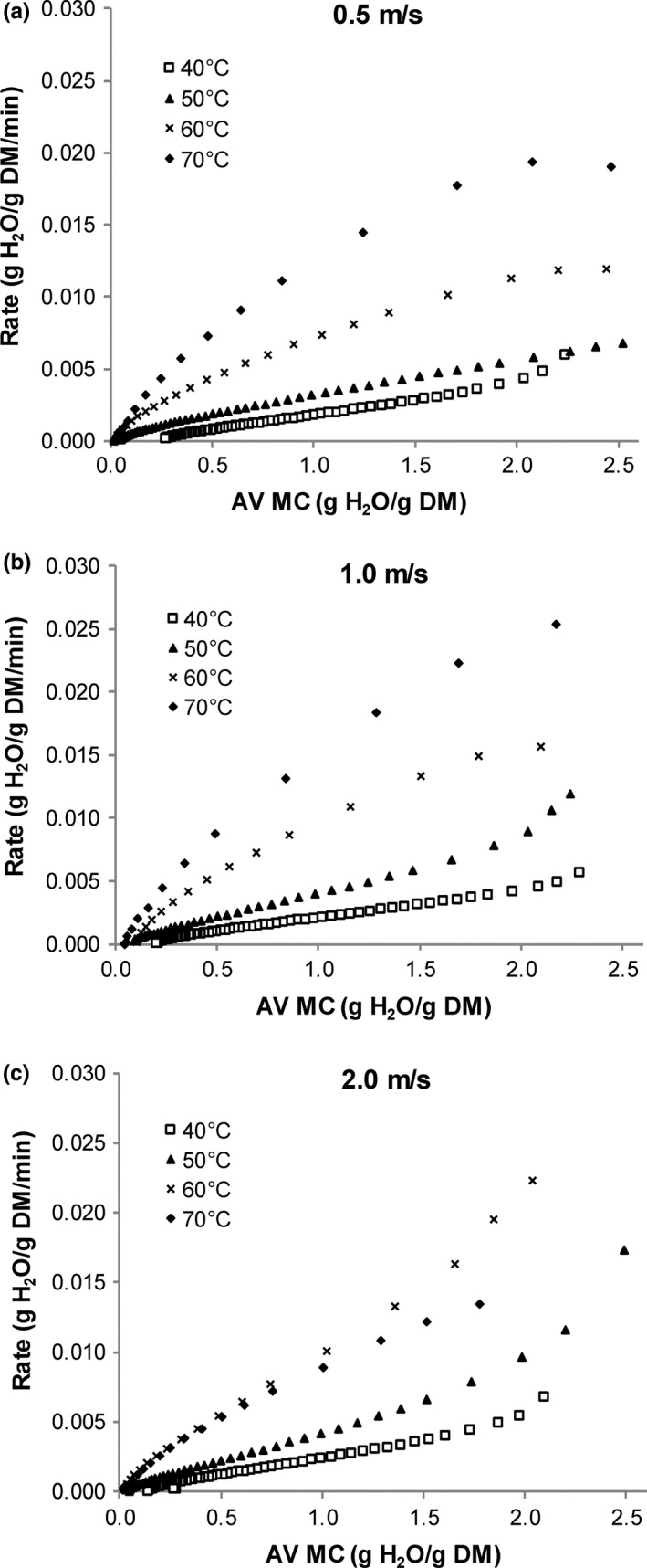
Drying rate as a function of average moisture content of lemongrass leaves dried at different air temperatures

Drying rate as a function of average moisture content at the different air velocities is shown in Figure 5a–c. With the exception of leaves dried at the highest air velocity of 2.0 m/s, increasing temperature from 40 to 70°C resulted in an increase in drying rate during the first 200 min. Drying was found to occur in the falling rate period. Leaves dried at the lowest air velocity of 0.5 m/s showed a short warm‐up period at the start of drying at 60 and 70°C, although not very pronounced. For leaves dried at 2.0 m/s, increasing air temperature to 70°C did not effect a further increase in drying rate.

As drying temperature increased from 40 to 70°C, the effect of increasing air velocity became more pronounced, especially at the higher moisture values. At 0.5 to 1.0 m/s, increasing temperature from 40 to 70°C resulted in an increase in drying rate. At the highest air velocity of 2.0 m/s, this trend was seen only as temperature increased from 40 to 60°C, with further increase in air velocity resulting in a small decrease in drying rate.

With respect to the effect of air velocity on drying rate at each temperature, a velocity effect was not apparent at 40°C. At drying temperatures of 50 and 60°C, increasing the air velocity from 0.5 to 1.0 m/s resulted in a noticeable increase in drying rate at higher moisture values in the range 1.0 to 3.0 g H_2_O/g DM. Further increase in the air velocity resulted in a further small increase in drying rate. For leaves dried at 70°C, increasing the air velocity beyond 1.0 m/s resulted in leaves having a lower drying rate.

With regard to the effect of temperature and air velocity, similar results were reported by Tzempelikos et al. (2014) for the drying of quince slices. They added that while an increase in temperature and velocity results in increased mass transfer, for large values of velocity, the temperature difference becomes more important while the effect of air velocity diminishes.

No similar work was found on drying rate versus moisture content for lemongrass leaves. Ibrahim et al. (2009) used *MR* curves as the basis to report the absence of a constant rate period at 35–55°C and 30–50% rh. Drying rate curves have been presented for other leafy materials such as spinach and drumstick leaves (Premi et al., 2010; Simha & Gugalia, 2013). Drying takes place by two mechanisms, internal moisture transfer, and surface moisture transfer. The constant rate period can be described as the initial stages of drying where the rate of internal moisture transfer is equal or greater than the rate of surface moisture transfer. Drying in the falling rate period occurs when the rate of internal moisture transfer is lower than the rate of surface moisture transfer. It is widely reported that drying of many agricultural materials occurs during the falling rate period only and that air velocity does not significantly affect drying rate as air velocity mainly affects the rate of external mass transfer and has little effect when internal diffusion is the limiting factor in the drying process (Sablani & Rahman, 2007). This study revealed that there was a velocity effect at the start of the drying process at 50 and 60°C.

Air movement is important particularly during the early stages of drying when the external mass transfer mechanism predominates, and during this time, air velocity will impact on the rate of external mass transfer. Erbay and Icier (2010) noted that the influence of drying air temperature was higher than that of air velocity in the drying of olive leaves, adding that the highest temperature and velocity combination did not give the highest drying rate in olive leaves dried at 50–70°C and 0.5 to 1.5 m/s. As seen in this study, higher heat transfer rates at 70°C and 2 m/s resulted in deleterious changes to the texture of the leaves which decreased the rate of moisture removal.

### 3.4 Drying rate constant (*k*) and effective diffusion coefficient (*D*_eff_)

The drying rate constants (*k*) were determined from the initial straight line portions (150–200 min) of plots of ln free moisture (ln *MR*) as a function of drying time (*t*) based on Equation (5). As given in Table 5, with the exception of leaves dried at 2 m/s at 70°C, *k‐*values increased as drying temperature increased (*p *≤* *.001) and there was a temperature‐velocity interaction effect (*p *≤* *.05). As temperature increased from 40 to 70°C, the *k*‐value increased from an average of 0.0144 to an average of 0.0457 1/min. Following the pattern seen for this data in earlier sections, *k*‐values were found to increase with air velocity at the lower temperatures of 40 and 50°C. Overall, the increase in *k‐*values was significant (*p *≤* *.05) as temperature was increased from 50 to 60 and then to 70°C, while the effect of air velocity was not significant. The *D*_eff_ values followed the same trend as the *k*‐values, increasing from 0.97 to 1.33 × 10^−10^ m^2^/s as temperature increased from 40 to 70°C at 0.5 m/s and increasing from 1.82 to 4.52 × 10^−10^ m^2^/s at 1.0 m/s.

**Table 5:** Drying rate constants (*k*) and diffusion coefficients (*D*_eff_) for dried lemongrass leaves

Air Temperature (°C)	Air Velocity (m/s)	*k* (1/min)	*R* ^*2*^	a *D*_eff_ (m^2^/s)
40	0.5	0.0023 ± 0.0002^e^	0.9994	0.97 × 10^−10^
	1.0	0.0025 ± 0.00005^e^	0.9994	1.04 × 10^−10^
	2.0	0.0028 ± 0.0003^e^	0.9994	1.16 × 10^−10^
50	0.5	0.0032 ± 0.0003^e^	0.9982	1.33 × 10^−10^
	1.0	0.0043 ± 0.0002^de^	0.9996	1.82 × 10^−10^
	2.0	0.0047 ± 0.0004^de^	0.9990	1.98 × 10^−10^
60	0.5	0.0074 ± 0.00005 ^cd^	0.9922	3.11 × 10^−10^
	1.0	0.0107 ± 0.0007^bc^	0.9943	4.52 × 10^−10^
	2.0	0.0107 ± 0.0004^bc^	0.9924	4.52 × 10^−10^
70	0.5	0.0140 ± 0.0027^b^	0.9924	5.90 × 10^−10^
	1.0	0.0190 ± 0.0033^a^	0.9924	8.01 × 10^−10^
	2.0	0.0104 ± 0.0015^bc^	0.9993	4.40 × 10^−10^

Values are means ± SEM, *n *= 2 per treatment group.

^a‐e^Means in a column without a common superscript letter differ (*p *< .05).

a *D*_eff_ = k (4L^2^/π^2^) where L = half thickness 0.25 cm.

For each drying temperature and velocity combination, the temperature dependence of the *D*_eff_ values and the activation energies were estimated from plots of ln *D*_eff_ versus *1/T* using an Arrhenius‐type equation:

(6) $$\text{In}D=-\frac{E_{a}}{\;\text{RT}}$$

A linear relationship was obtained for leaves dried at 1 m/s at 1.0 m/s, and the *E*_*a*_ value was calculated to be 62,476 J/mol (*R* ^*2*^ = 0.9893). At air velocities of 0.5 and 2 m/s, the slopes were not found to be linear for the range of temperatures (40–70°C). For these cases, the slopes were taken for the straight line portion only. For the lowest air velocity of 0.5 m/s, the slope became linear in the range of 50–70°C, and the activation energy determined to be 67,917 J/mol (*R* ^*2*^ = 0.9949). At the highest air velocity of 2 m/s, the slope was linear at the temperature range of 40–60°C, with the *E*_*a*_ value of 58,894 J/mol (*R* ^*2*^ = 0.9812).

No parallel works for lemongrass have reported on rate constant determination and calculation of effective moisture diffusivity (*D*_eff_). The drying constant is said to be dependent on the material properties and the characteristics of the drying air as it represents several transport phenomena (Mujumdar, 2007). It is generally expected that as the drying rate increases, the drying rate constant will also increase, as happens with an increase in temperature. Diffusion of moisture controls the rate of drying in the falling rate period. An increase in the effective diffusivity is an indicator of lower resistance to mass transfer in the material dried. The diffusivity of water or water vapor of a material during drying is dependent on its structure or porosity and temperature (Naidu et al., 2012).

Coradi et al. (2014) calculated diffusion coefficients of lemongrass leaves based on the constants (*k*) obtained from fitting the data to the two‐term model and found values to increase from 2.5 to 4.5 × 10^−11 ^m^2^/s as temperature increased from 40 to 70°C. For other leafy materials, researchers have also reported an increase in diffusions coefficients with increasing temperature in mint, bay leaves, olive leaves, spinach, verbena, and fever leaves with values in the order of 10^−12^ to 10^−8^ m^2^/s (Barbosa et al., 2007; Cakmak et al., 2013; Doymaz, 2006, 2014; Erbay & Icier, 2010; Premi et al., 2010; Simha & Gugalia, 2013; Sobukola & Dairo, 2007). Activation energy values for leaves such as mint, olive, and bay leaves have ranged between 31.79 and 62.93 kJ/mol (Barbosa et al., 2007; Cakmak et al., 2013; Doymaz, 2006, 2014; Erbay & Icier, 2010).

### 3.5 Moisture ratio and thin‐layer models

**Figure 6:**
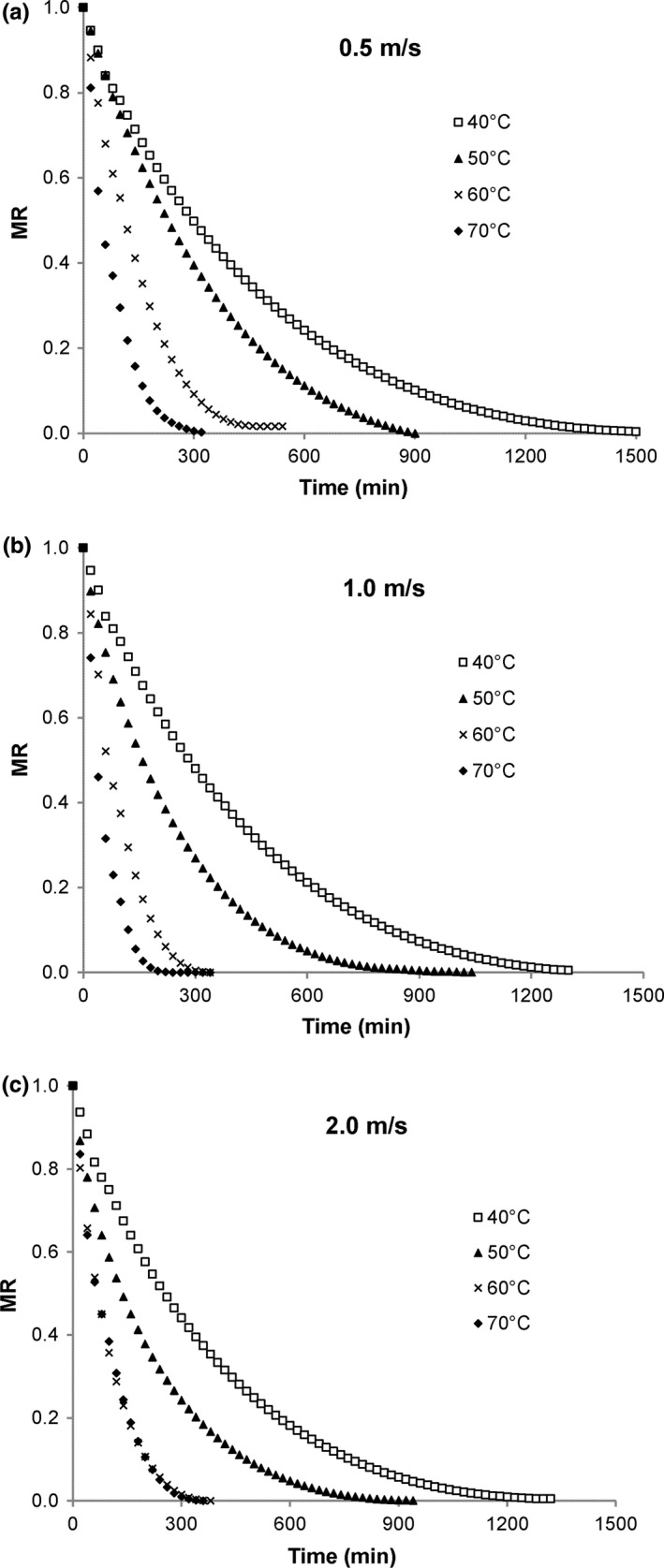
Moisture ratio curves for lemongrass leaves dried at different air velocities

Moisture ratio (*MR*) calculated based on Equation (4) plots are given in Figure 6a–c. Moisture ratio values were significantly affected by drying time, temperature, and a time–temperature interaction (*p *≤* *.001). As seen previously for the drying curves, increase in temperature resulted in increased decline in *MR* values, with the exception of leaves dried 2 m/s at 70°C.

With respect to lemongrass, Ibrahim et al. (2009) presented *MR* curves for leaves dried at 35–55°C and 30–50% rh, reporting that the main factor influencing drying to be temperature. Coradi et al. (2014) presented MR plots for lemongrass leaves dried at 40–70°C showing a temperature effect beyond 60 min of drying. Moisture ratio curves given by Kadam et al. (2011) for mint leaves revealed a trend of increasing decline in *MR* as temperature increased.

Of the twenty‐two thin‐layer models applied to the MR data, the coefficients for the five models which best fit the drying data obtained at 40–70°C and 0.5 to 2.0 m/s are given in Table 6 a to c. Model fit couple be expected to differ because the shapes of the *MR* curves for leaves dried under different conditions of temperature and velocity also differ. Beyond the models that best fit the data, the other models showed regression coefficients of <0.900 and some models failed.

**Table 6:** Thin‐layer model fit and constants for top models for lemongrass leaves

Temp	Model	Model constant								*R* ^*2*^	*RMSE*	χ^*2*^
		*k*	*n*	*a*	*b*	*k*_*1*_	*g*	*k*_*2*_	*a*_*1*_			
a) Air velocity: 0.5 m/s												
40°C	Alibas	1.0261	1.0000	1.0281	1.0240	–	−0.0436	–	–	0.9996	0.004988	0.000027
	Midilli	0.0019	1.0248	0.9814	0.0000	–	–	–	–	0.9995	0.005643	0.000034
	Aghbashlo et al.	–	–	–	–	0.0022	–	−0.0002	–	0.9988	0.009328	0.000089
	Peleg	–	–	357.98	0.7382	–	–	–	–	0.9965	0.015641	0.000251
	Wang & Singh	–	–	−0.0016	0.0000	–	–	–	–	0.9733	0.043182	0.001913
50°C	Midilli	0.0019	1.0757	0.9935	−0.0001	–	–	–	–	0.9999	0.002468	0.000007
	Aghbashlo et al.	–	–	–	0.0000	0.0027	–	−0.0005	–	0.9995	0.006358	0.000042
	Peleg	–	–	306.16	0.6320	–	–	–	–	0.9979	0.012844	0.000173
	Wang & Singh	–	–	−0.0024	0.0000	–	–	–	–	0.9957	0.018624	0.000363
	Alibas	1.5084	0.9994	1.3218	1.4998	–	−0.1619	–	–	0.9751	0.044666	0.002244
60°C	Aghbashlo et al.	–	–	–	–	0.0056	–	−0.0009	–	0.9992	0.008344	0.000075
	Logistic	0.0101	–	–	–	–	–	–	1.7932	0.9987	0.010395	0.000122
	Midilli	0.0024	1.1939	0.9760	0.0000	–	–	–	–	0.9982	0.012377	0.000180
	Alibas	1.0008	1.0009	1.0022	0.9991	–	−0.0207	–	–	0.9980	0.012907	0.000204
	Modified Page	0.0067	1.1758	–	–	–	–	–	–	0.9975	0.014509	0.000227
70°C	Midilli	0.0122	1.0142	1.0061	−0.0001	0.0000	–	–	–	0.9976	0.014858	0.000294
	Aghbashlo et al.	0.0000	0.0000	0.0000	–	0.0123	–	−0.0007	–	0.9973	0.015629	0.000279
	Modified Page	0.0131	1.0586	0.0000	–	–	–	–	–	0.9971	0.016321	0.000304
	Page	0.0102	1.0586	0.0000	–	–	–	–	–	0.9971	0.064723	0.004788
	Logistic	0.0148	0.0000	4.7884	–	–	–	–	5.8152	0.9970	0.016526	0.000336
b) Air velocity: 1.0 m/s												
40°C	Alibas	1.0618	1.0001	1.0220	1.0602	–	−0.0397	–	–	0.9995	0.005873	0.000037
	Midilli	0.0015	1.0752	0.9787	0.0000	–	–	–	–	0.9994	0.006457	0.000044
	Aghbashlo et al.	–	–	–	–	0.0023	–	−0.0002	–	0.9991	0.007830	0.000063
	Peleg	–	–	340.2111	0.7271	–	–	–	–	0.9948	0.019458	0.000389
	Wang & Singh	–	–	–−0.0018	0.0000	–	–	–	–	0.9791	0.038952	0.001559
50°C	Alibas	1.0041	0.9999	1.0166	0.9996	–	–0.0247	–	–	0.9994	0.006393	0.000045
	Midilli	0.0035	1.0352	0.9802	0.0000	–	–	–	–	0.9993	0.006618	0.000047
	Aghbashlo et al.	–	–	–	–	0.0042	–	−0.0002	–	0.9991	0.007848	0.000064
	Page	0.0035	1.0464	0.0000	–	–	–	–	–	0.9982	0.010875	0.000123
	Modified Page	0.0045	1.0464	0.0000	–	–	–	–	–	0.9982	0.010875	0.000123
60°C	Aghbashlo et al.	–	–	–	–	0.0087	–	−0.0014	–	0.9984	0.012612	0.000180
	Midilli	0.0063	1.1001	1.0001	−0.0001	–	–	–	–	0.9984	0.012592	0.000207
	Alibas	0.9030	1.0008	1.0422	0.8969	–	−0.0411	–	–	0.9984	0.012306	0.000215
	Modified Page	0.0104	1.1682	–	–	–	–	–	–	0.9970	0.017055	0.000330
	Logistic	0.0148	–	1.1337	–	–	–	–	2.1235	0.9971	0.016644	0.000336
70°C	Hii	0.0002	1.8998	0.4857	–	–	0.0020	–	–	0.9998	0.004258	0.000031
	Page	0.0120	1.1090	–	–	–	–	–	–	0.9976	0.015348	0.000283
	Modified Page	0.0185	1.1090	–	–	–	–	–	–	0.9976	0.015348	0.000283
	Aghbashlo et al.	–	–	–	–	0.0170	–	−0.0012	–	0.9973	0.016360	0.000321
	Alibas	1.1799	1.0013	1.0139	1.1676	–	−0.0090	–	–	0.9978	0.014601	0.000365
	Midilli	0.0136	1.0768	1.0061	–	–	–	–	–	0.9978	0.014617	0.000321
c) Air velocity: 2.0 m/s												
40°C	Midilli	0.0023	1.0209	0.9820	0.0000	–	–	–	–	0.9995	0.005861	0.000037
	Alibas	1.1841	1.0000	1.0271	1.1816	–	−0.0424	–	–	0.9996	0.005259	0.000030
	Aghbashlo et al.	–	–	–	–	0.0026	–	−0.0002	–	0.9988	0.009106	0.000086
	Peleg	–	–	303.62	0.7386	–	–	–	–	0.9966	0.015347	0.000243
	Wang & Singh	–	–	−0.0020	0.0000	–	–	–	–	0.9744	0.042330	0.001848
50°C	Alibas	1.0037	0.9994	1.0348	0.9962	–	−0.0435	–	–	0.9996	0.005139	0.000030
	Midilli	0.0078	0.9080	0.9872	0.0000	–	–	–	–	0.9995	0.005404	0.000032
	Modified Page	0.0050	0.9542	–	–	–	–	–	–	0.9975	0.012384	0.000160
	Page	0.0064	0.9542	–	–	–	–	–	–	0.9975	0.012384	0.000160
	Peleg	–	–	154.20	0.8083	–	–	–	–	0.9975	0.012439	0.000162
60°C	Midilli	0.0096	1.0130	0.9946	−0.0001	–	–	–	–	0.9996	0.005996	0.000046
	Aghbashlo et al.	–	–	–	–	0.0097	–	−0.0007	–	0.9992	0.008324	0.000077
	Logistic	0.0128	–	2.4118	–	–	–	–	3.3633	0.9985	0.011167	0.000148
	Modified Page	0.0106	1.0667	–	–	–	–	–	–	0.9982	0.012557	0.000176
	Page	0.0079	1.0667	–	–	–	–	–	–	0.9982	0.012557	0.000176
70°C	Midilli	0.0100	0.9972	1.0003	−0.0001	–	–	–	–	0.9979	0.013762	0.000244
	Alibas	1.5084	0.9999	1.0570	1.4982	–	−0.0558	–	–	0.9980	0.013259	0.000243
	Aghbashlo et al.	–	–	–	–	0.0093	–	−0.0009	–	0.9971	0.016131	0.000293
	Logistic	0.0128	–	2.1489	–	–	–	–	3.1148	0.9958	0.019353	0.000449
	Peleg	–	–	82.91	0.7328	–	–	–	–	0.9956	0.019819	0.000442

**Figure 7:**
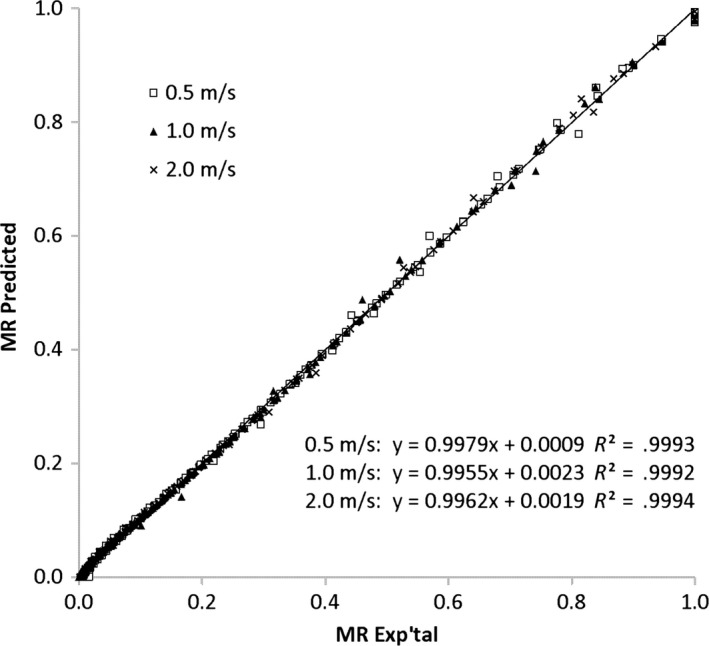
Comparison of predicted versus experimental moisture ratio values for lemongrass leaves dried at 50°C using the Midilli model

Although model fit differed with the drying conditions of temperature and air velocity, the Midilli model could be applied to all data with reasonable prediction of *MR* values as shown in Figure 7 for the drying data at 50°C at 0.5 to 1.5 m/s. Other models which best fit the data in this study included the Alibas, Aghbashlo et al., and the Hii model for the *MR* data for leaves dried at the highest air velocity of 2.0 m/s at the highest temperature of 70°C. For leaves dried at 1.0 m/s, the Midilli model best fits the data for leaves at 40, 60, and 70°C and was a close second to the Alibas model (derived from the Midilli model) for leaves dried at 50°C. Kemat et al. (2008) who dried lemongrass leaves in a fluidized bed dryer (temperature 30–90°C, air velocity 0.873–1.091 m/s, bed height 1–4 cm) also reported the Midilli model to best fit the drying data.

Other studies on lemongrass leaves have reported model fit to differ with drying method. Waewsak et al. (2006) found the Wang and Singh model to best describe the drying data for lemongrass leaves dried in a biomass dryer at 60°C, of the thirteen models tested. Ibrahim et al. (2009) found the Newton model to best fit the data for lemongrass leaves dried in a constant temperature and humidity chamber at three drying temperatures (35, 45, 55°C) and three relative humidity conditions (30, 40, and 50%) at a fixed air velocity of 1 m/s. Coradi et al. (2014) reported that the two‐term model best fits the drying data (40–70°C) for cut leaves dried in a fixed bed dryer.

In general, thin‐layer model fit varies widely with respect to leafy materials and is found to depend on drying method and temperature. The Midilli model has been reported to best describe the drying data for bay leaves, verbena leaves microwave‐dried spinach leaves (Barbosa et al., 2007; Cakmak et al., 2013; Doymaz, 2014; Simha & Gugalia, 2013). The Page model has been reported to best describe the *MR* data for hot air‐dried bay leaves, coriander, fever leaves, and spinach (Gunhan, Demir, Hancioglu, & Hepbasli, 2005; Shaw et al., 2007; Simha & Gugalia, 2013; Sobukola & Dairo, 2007) while the Verma, logarithmic, and two‐term models were used in other studies (Doymaz, 2006; Kadam et al., 2011; Premi et al., 2010). Erbay and Icier (2010) reported that the modified Henderson and Pabis model best fits the *MR* data for olive leaves dried at 50 to 70°C at 0.5 to 1.5 m/s.

## 4 CONCLUSIONS

Increasing temperature from 40 to 60°C resulted in a dramatic decrease in total drying time, increase in drying rate, and decrease in equilibrium moisture content of leaves. Increasing temperature to from 40 to 60°C resulted in an average decrease in total drying time of 71%. The effect of air velocity was more important during the initial stages of drying, and insignificant at lower moisture values. Additionally, drying of leaves at higher velocities of 2.0 m/s is not recommended as the leaves blow about as they dry and become lighter. Drying at all temperatures and velocity combinations took place in the falling rate period. Overall, the average equilibrium moisture values of leaves dried at 40°C were approximately 35% higher than the average value for leaves dried at 50°C and 80% higher than the average value for leaves dried at 60°C. Increasing temperature from 40 to 60°C resulted in an average decrease in total drying time of 71%, while further increasing the temperature to 70°C resulted in an average decrease of 78%. Drying rate constants and diffusivity values were successfully determined. Model fit varied according to specific temperature–velocity combination, with the Midilli model adequately describing the *MR* data for the range of temperature and air velocity treatments. *D*_eff_ values ranged from 0.97 to 8.01 × 10^−10^ m/s. To achieve a final moisture content value of 11% (wb), lemongrass leaves were dried for 725 min at 50°C (1 m/s). Leaves dried at 40°C did not achieve this moisture value when dried at 0.5 and 1.0 m/s, with the equilibrium moisture values averaging between 18 and 20% (wb). Leaf quality in terms of subjective ratings was adversely affected at temperatures above 50°C. As the best possible combination of drying temperature and air velocity based on drying time and overall quality attributes, drying of lemongrass leaves at 50°C is recommended at a velocity of 1 m/s, as further increasing the air velocity to 2 m/s will not improve drying time.

## CONFLICT OF INTEREST

None declared.

**NOMENCLATURE**
*A*: Drying constant
*a*_*w*_: Water activity
*db*: Dry basis (g H_2_O/100 g DM)
*D*_eff_: Diffusion coefficient (m^2^/s)
*DM*: Dry matter (g)
*E*_*a*_: Activation energy (J/mol)
*FW*: Fresh weight (g)
*k, k*_*1*_*,a, a*_*1*_*,b,g,n*: Model constants
*k*: Drying rate constant (1/min)
*L*: Half thickness of sample (cm)
*M*_e_: Equilibrium moisture content (g H_2_O/g DM)
*M*: Moisture content (g H_2_O/g DM) at time = t
*M*_o_: Initial moisture content (g H_2_O/g DM)
*MR*: Moisture ratio
*R*^*2*^: Coefficient of determination
R: Gas constant (8.314 J/Kmol),
RMSE: Root mean square error
*T*: Process temperature (K)
*t*: Time (min)
*wb*: Wet basis (g H_2_O/100 g FW)
χ^2^: Chi‐square

## References

[fsn3642-bib-0001] Aghbashlo, M. , Kianmehr, M. H. , & Hassan‐Beygi, S. R. (2010). Drying and rehydration characteristics of sour cherry (*Prunus cerasus* L.). Journal of Food Processing and Preservation, 34, 351–365.

[fsn3642-bib-0002] Ahmed, J. , Shivhare, U. S. , & Singh, G. (2001). Drying characteristics and product quality of coriander leaves. Food and Bioproducts Processing, 79, 103–106. https://doi.org/10.1205/096030801750286258

[fsn3642-bib-0003] Alibas, I. (2014). Microwave, air and combined microwave‐air drying of grape leaves (*Vitis vinifera* L.) and the determination of some quality parameters. International Journal of Food Engineering, 10, 69–88.

[fsn3642-bib-0004] Barbosa, F. F. , Melo, E. C. , Santos, R. H. S. , Da Rocha, R. P. , Martinazzo, A. P. , Raunz, L. L. , & Gracia, L. M. (2007). Evaluation of mathematical models for prediction of thin‐layer drying of Brazilian lemon‐scented verbena leaves (*Lippia alba* [Mill] N.E.Brown). Produtos Agroindustriais, 9, 71–80.

[fsn3642-bib-0501] Buggle, V. , Ming, L. C. , Furtado, E. L. , Rocha, S. F. R. , & Marques, M. O. M. (1999). Influence of different drying temperatures on the amount of essential oils and citral content in cymbopogon citratus (dc) stapf‐poaceae. Acta Horticulturae (ISHS), 500, 71–74.

[fsn3642-bib-0005] Cakmak, H. , Kumcuoglu, S. , & Tavman, S. (2013). Thin layer drying of bay leaves (*Laurus nobilis* L.) in conventional and microwave oven. Akademik Gıda, 11, 20–26.

[fsn3642-bib-0007] Chen, X. D. , & Patel, K. C. (2008). Biological changes during food drying processes In Chen X. D., & Mujumdar A. S. (Eds.), Drying technologies in food processing (pp. 90–112). West Sussex, UK: Blackwell Publishing Ltd.

[fsn3642-bib-0006] Coradi, P. C. , Melo, E. C. , & Rocha, R. P. (2014). Mathematical modeling of the drying kinetics of the leaves of lemon grass (*Cymbopogon citratus* Stapf) and its effects on quality. Idesia (Arica), 32, 43–56. https://doi.org/10.4067/S0718-34292014000400006

[fsn3642-bib-0502] Daniel, M. (2006). Medicinal plants‐chemistry and properties. Enfield, NH: Science Publishers.

[fsn3642-bib-0008] Demir, V. , Gunhan, T. , Yagcioglu, A. K. , & Degirmencioglu, A. (2004). Mathematical modelling and the determination of some quality parameters of air‐dried bay leaves. Biosystems Engineering, 88, 325–335. https://doi.org/10.1016/j.biosystemseng.2004.04.005

[fsn3642-bib-0009] Doymaz, I. (2006). Thin‐layer drying behavior of mint leaves. Journal of Food Engineering, 74, 370–375. https://doi.org/10.1016/j.jfoodeng.2005.03.009

[fsn3642-bib-0010] Doymaz, I. (2014). Thin‐layer drying of bay laurel leaves (*Laurus nobilis* L.). Journal of Food Processing and Preservation, 38, 449–456. https://doi.org/10.1111/j.1745-4549.2012.00793.x

[fsn3642-bib-0011] Erbay, Z. , & Icier, F. (2010). Thin‐layer drying behaviors of olive leaves (*Olea europaea* L.). Journal of Food Process Engineering, 33, 287–308. https://doi.org/10.1111/(ISSN)1745-4530

[fsn3642-bib-0012] Fudholi, A. , Ruslan, M. H. , Othman, M. Y. , Saadatian, O. , Zaharim, A. , & Sopian, K. (2012). Investigation of Medical Herbs Moisture in Solar Drying. in: Advances in Environment, Biotechnology and Biomedicine, 1st WSEAS International Conference on Agricultural Science, Biotechnology, Food and Animal Science (ABIFA ‘12), Tomas Bata University in Zlin, Czech Republic, Sept 20‐22, 2012. Wseas LLC., Czech Republic, pp. 127–131.

[fsn3642-bib-0013] Gunhan, T. , Demir, V. , Hancioglu, E. , & Hepbasli, A. (2005). Mathematical modelling of drying of bay leaves. Energy Conversion and Management, 46, 1667–1679. https://doi.org/10.1016/j.enconman.2004.10.001

[fsn3642-bib-0503] Hunterlab (2008). CIE L*a*b* Color Scale. Applications note, insight on color 8(7). Reston, VA: Hunter Associates Laboratory Inc.

[fsn3642-bib-0015] Hyams, D. G. (2016). CurveExpert software. Available from: http://www.curveexpert.net. [last accessed 02.02.2016].

[fsn3642-bib-0016] Ibrahim, M. , Sopian, K. , & Daud, W. R. (2009). Study of the drying kinetics of lemongrass. American Journal of Applied Sciences, 6, 1070–1075.

[fsn3642-bib-0017] Kadam, D. M. , Goyal, R. K. , Singh, K. K. , & Gupta, M. K. (2011). Thin layer convective drying of mint leaves. Journal of Medicinal Plants Research, 5, 164–170.

[fsn3642-bib-0018] Kassem, A. M. , El‐Batawi, I. E. , & Sidky, M. M. A. (2006). Effect of Solar Energy and Other Drying Methods on Quality Characteristics of Some Medicinal Plants. The 14th. Annual Conference of the Misr Society of Ag. Eng. (MSAE), 22‐23 Nov. 2006, pp.760‐776.

[fsn3642-bib-0019] Kemat, S. A. A. , Rahman, N. A. , & Wahit, R. (2008). “Determination of suitable thin layer drying curve model for lemongrass (Cymbopogan citrates)”. RSCE‐SOMCHE 27th Symposium of Malaysian Chemical Engineers (SOMChE 2014) and 21st Regional Symposium on Chemical Engineering (RSCE 2014). Addressing the Grand Challenges of Chemical Engineers in the 21st Century. Taylor's University Lakeside Campus, Selangor Darul Ehsan, Malaysia, 29‐30 October 2014, pp.945‐948.

[fsn3642-bib-0020] Konica Minolta Sensing Inc . (2003). “Precise color communication”. Part IV: Color Terms. Available from: http://www2.konicaminolta.eu/eu/Measuring/pcc/en/part4/05.html [last accessed 09.02.2016].

[fsn3642-bib-0021] Kucuk, H. , Midilli, A. , Kilic, A. , & Dincer, I. (2014). A review on thin‐layer drying‐curve equations. Drying Technology, 32, 757–773. https://doi.org/10.1080/07373937.2013.873047

[fsn3642-bib-0022] Lonkar, P. B. , Chavan, U. D. , Pawar, V. D. , Bansode, V. V. , & Amarowicz, R. (2013). Studies on preparation and preservation of lemongrass (*Cymbopogon flexuosus* (Steud) wats) powder for tea. Emirates Journal of Food and Agriculture, 25, 585–592. https://doi.org/10.9755/ejfa.

[fsn3642-bib-0023] Martinazzo, A. P. , Melo, E. C. , Barbosa, L. C. de. A. , Soares, N. de. F. F. , Rocha, R. P. , Randüz, L. L. , & Berbert, P. A. (2009). Quality parameters of *Cymbopogon citratus* leaves during ambient storage. Applied Engineering in Agriculture, 25, 543–547. https://doi.org/10.13031/2013.27457

[fsn3642-bib-0024] Mujaffar, S. , & Sankat, C. K. (2005). The air drying behavior of shark fillets. Canadian Biosystems Engineering, 47, 11–21.

[fsn3642-bib-0025] Mujaffar, S. , & Sankat, C. K. (2015). Modeling the drying behavior of unsalted and salted catfish (*Arius* sp.) Slabs. Journal of Food Processing and Preservation, 39, 1385–1398. https://doi.org/10.1111/jfpp.12357

[fsn3642-bib-0026] Mujumdar, A. S. (2007). Handbook of industrial drying (pp. 99–100). Boca Raton, FL: CRC Press.

[fsn3642-bib-0027] Naidu, M. , Khanum, H. , Sulochanamma, G. , Sowbhagya, H. , Hebbar, U. , Prakash, M. , & Srinivas, P. (2012). Effect of drying methods on the quality characteristics of fenugreek (*Trigonellafoenum‐graecum*) greens. Drying Technology, 30, 808–816. https://doi.org/10.1080/07373937.2012.666607

[fsn3642-bib-0029] Premi, M. , Sharma, H. , & Upadhyay, A. (2010). Effect of air velocity and temperature on the drying kinetics of drumstick leaves (*Moringa Oleifera*). International Journal of Food Engineering, 8, 391–400.

[fsn3642-bib-0030] Rahman, N. A. , Tasirin, S. M. , Razak, A. H. A. , Mokhtar, M. , & Muslim, S. (2013). Comparison of drying parameter optimization of lemon grass. World Applied Sciences Journal, 24, 1234–1249.

[fsn3642-bib-0031] Rodríguez, J. , Clemente, G. , Sanjuán, N. , & Bon, J. (2014). Modelling drying kinetics of thyme (*Thymus vulgaris* L.): Theoretical and empirical models, and neural networks. Food Science and Technology International, 20, 13–22. 23733820 10.1177/1082013212469614

[fsn3642-bib-0032] Sablani, S. S. , & Rahman, M. S. (2007). Fundamentals of Food Drying In Hui Y. U. (Ed.), Food drying: Science and technology (pp. 1–42). Lancaster, PA: DEStech.

[fsn3642-bib-0033] Sanmeema, N. , Poomsa‐ad, N. , & Wiseet, L. (2012). Lemongrass drying under hot air and nitrogen as drying medium at different drying temperatures. Journal of Science and Technology MSU, 31, 650–654.

[fsn3642-bib-0034] Shaw, M. , Meda, V. , Tabil, J. , & Opoku, A. (2007). Drying and color characteristics of coriander foliage using convective thin ‐layer and microwave drying. Journal of Microwave Power and Electromagnetic Energy, 41, 56–65. 18161423

[fsn3642-bib-0035] Silva, W. P. , Silva, C. M. D. P. S. , Gama, F. J. , & Gomes, J. P. (2014). Mathematical models to describe thin‐layer drying and to determine drying rate of whole bananas. Journal of the Saudi Society of Agricultural Sciences, 13, 67–74. https://doi.org/10.1016/j.jssas.2013.01.003

[fsn3642-bib-0036] Simha, P. , & Gugalia, A. (2013). Thin layer drying kinetics and modelling of Spinacia oleracea leaves. International Journal of Applied Engineering Research, 8, 1053–1066.

[fsn3642-bib-0037] Sobukola, O. P. , & Dairo, O. U. (2007). Modeling drying kinetics of fever leaves (*Ocimum viride*) in a convective hot air dryer. Nigerian Food Journal, 25, 146–154.

[fsn3642-bib-0038] Tzempelikos, D. A. , Vouros, A. P. , Bardakas, A. V. , Filios, A. E. , & Margaris, D. P. (2014). Case studies on the effect of the air drying conditions on the convective drying of quinces. Case Studies in Thermal Engineering, 3, 79–85. https://doi.org/10.1016/j.csite.2014.05.001

[fsn3642-bib-0504] VSN International Limited (2014). GenStat for windows discovery edition 4 software release 10.3. Hemel Hempstead, UK.

[fsn3642-bib-0040] Waewsak, J. , Chindaruksa, S. , & Punlek, C. (2006). A mathematical modeling study of hot air drying for some agricultural products. Thammasat International Journal of Science and Technology, 11, 14–20.

